# Warming and cooling effects of local climate zones on urban thermal environment

**DOI:** 10.3389/fpubh.2022.1072174

**Published:** 2022-11-23

**Authors:** Rui Zhang, Jun Yang, Dongqi Sun, Xinyue Ma, Wenbo Yu, Xiangming Xiao, Jianhong (Cecilia) Xia

**Affiliations:** ^1^Urban Climate and Human Settlements Research' Lab, Jangho Architecture College, Northeastern University, Shenyang, China; ^2^School of Humanities and Law, Northeastern University, Shenyang, China; ^3^Human Settlements Research Center, Liaoning Normal University, Dalian, China; ^4^Key Laboratory of Regional Sustainable Development Modeling, Institute of Geographic Sciences and Natural Resources Research, CAS, Beijing, China; ^5^Department of Microbiology and Plant Biology, Center for Earth Observation and Modeling, University of Oklahoma, Norman, OK, United States; ^6^School of Earth and Planetary Sciences (EPS), Curtin University, Perth, WA, Australia

**Keywords:** land surface temperature, local climate zone, stepwise regression model, urban thermal characteristics, Shenyang

## Abstract

Understanding the thermal characteristics and contribution ranking of local climate zones (LCZs) is essential since they can help in maintaining environmental harmony. However, previous studies only considered independent effects and could not analyze the combined effects of LCZ on land surface temperature (LST). In this study, we propose a new method to establish an interaction model between LCZs. Five first-level grids with different scales from 270 to 990 m were established to calculate the area proportion of LCZ. The area proportion of LCZ was then applied in the stepwise regression model to quantitatively analyze its magnitude and direction of impact on the LST. The results suggest that the LCZ types of the study area with the highest and lowest average LST were LCZ2 (compact middle-rise building, 39.82°C) and LCZG (water body, 34.24°C), respectively. However, on most scales, the warming effect of LCZ2 was lower than that of LCZE (bare rock or paver), and the cooling effect of LCZG was lower than that of LCZD (low plants). The optimum results were obtained at a scale of 810 m. At this scale, the warming effect was in the order: LCZE (0.314) > LCZ2 (0.236) > LCZ3 (compact low-rise building, 0.135) > LCZ5 (open middle-rise, 0.084) > LCZ6 (open low-rise, 0.056); the cooling effect was in the order: LCZD (−0.272) > LCZA (dense trees, −0.104) > LCZG (−0.103). These findings can help to elucidate the unique warming and cooling effects of LCZ on the interaction condition and the construction of an urban human settlement.

## Introduction

With the continued progress of urbanization, artificial structures such as roads and houses are increasing in number and substantially changing the surface form (1). Extensive urban growth in the past has created a particular burden on the environment, thereby affecting the balance of urban climate regulation and causing numerous problems such as the heat island effect and air pollution (2–4). Global heat exposure has risen dramatically in recent years, thus increasing the risk of extreme heat and the frequency of deaths; heat-related excess deaths are projected to rise by 2.4% in 2030 and by 5.5% in 2090 (5–9). To protect human life, the following goals have been adopted by researchers worldwide, namely: improving production, increasing living efficiency, reducing energy consumption (10–13), maintaining an ecological balance, and creating a comfortable and healthy urban thermal environment (14–16).

Land surface temperature (LST), which refers to the temperature at the intersection of the land surface and the atmosphere, affects many natural ecological processes including atmospheric circulation and energy balance. It is a critical parameter for monitoring the urban thermal environment. Remote sensing technology can provide thermal radiation information, as well as multi-temporal and synchronous LST data over large areas, for thermal environment research (17). For example, surface temperature data can be used to analyze the intensity and distribution characteristics of heat islands at different temporal and spatial scales to coordinate ecological protection and urban construction processes (18–21).

The mechanisms underlying LST change are complex. Researchers often consider the effects of urban form and landscape on LST (22–25). Variations in land surface cover often lead to temperature differences. The LSTs of natural covers such as grassland and water are usually lower than those of built-up areas (21). To quantitatively analyze the factors influencing LST, the normalized vegetation index (NDVI) and normalized water body index (NDWI) are calculated. A positive correlation has been found between the percentage of impervious surface and LST (26). The influence of factors such as building density (BD), average building height (BH), and floor area ratio (FAR) on the urban thermal environment has also been widely studied (14, 27–29). The correlation between BH and LST is weak in the daytime, but strongly positive at night. The aspect ratio of street canyons in urban core areas is negatively correlated with LST in the daytime and positively correlated during nighttime (30). Socio-economic development and human activities also affect the urban thermal environment. Based on the perspective of urban functional areas, Chen et al. (31) noted that the thermal contributions of residential, industrial, and commercial service facility lands vary significantly. However, the contribution of anthropogenic heat to the urban thermal environment is relatively weak, while solar radiation, surface type, and urban form play more critical roles (32).

To better describe the impact of urban land cover, surface morphology, and three-dimensional architectural features on the thermal environment, Oke and Stewart (33) proposed the concept of local climate zone (LCZs). This concept has been widely used (34–36) as a highly versatile urban form zoning method, which can easily analyze urban characteristics and compare multiple cities under a unified standard (37, 38).

There are numerous studies on the thermal environment using the LCZ concept (39–42). Considering the calculation of heat island intensity as an example, many limitations exist in the traditional urban–rural binary division. The introduction of LCZ improves the fuzzy division of the previous binary structure, and reduces the difficulty in analyzing heat island characteristics. The temperature differences between LCZ classes are used to better describe the spatial differentiation characteristics of heat islands, which lays a foundation for further analysis of the driving force of heat island intensity (43). Several studies have analyzed the temperature differences and characteristics within and between LCZ classes and found that the LST of building types was generally higher than that of natural types (44, 45). From the LCZ perspective, LST exhibits obvious day–night and seasonal differences (46, 47). Chang et al. (26) classified LCZ by community units and analyzed the diurnal variation characteristics of LST on different types of LCZ in downtown Xi'an by combining ECOSTRESS (ECOsystem Spaceborne Thermal Radiometer Experiment on Space Station) data. These results showed that the warming rate of low-rise compact buildings was higher than that of open high-rise buildings in the daytime, and the height effect became insignificant during nighttime (26). The influence of LCZ on the thermal environment varies in cities of different sizes; the occurrence probability is higher in large cities (48). For the impact of specific types, the subjective thermal perception of “warm” was found to be more likely observed in LCZ types with close high-rise buildings (39).

These previous studies on the impacts of different types of LCZs on LST are mostly limited to the thermal differences, and the combined effect from different LCZ types is generally ignored (49). Although some studies quantify the relationship between LCZ and LST from a statistical perspective, the connection between LCZ and LST is independent (40, 45). All these studies were based on the hypothesis that when exploring the relationship between a certain type of LCZ and LST, the changes in other LCZ types are independent and do not affect the results. This hypothesis is reasonable because changes in LCZ do not occur dramatically in a short time and they are in a relatively static state. However, it may neglect the interaction of different types of LCZ in space, and it failed to analyze how different types of LCZs synchronously affect the LST. There have been many studies using land use for reference. For example, in addition to calculating the independent impact of each factor on LST, the land use and vegetation cover types of adjacent plots have a specific interaction with the ontology, thereby affecting the changes in LST (50, 51). Given that the competition of land use types affects the urban LST, which can be calculated using regression models, this study assumed that the interaction between different LCZs is also likely to affect the changes in surface temperature. Other than calculating the effect produced by a single LCZ type, we quantified the combined effect of all LCZs on the LST.

Herein, we have created a new method to analyze the combined effects of LCZ. Firstly, we classified LCZ and calculated LST to evaluate urban thermal characteristics from the traditional perspective. Next, we created a first-level grid as a platform to analyze the joint impact of the LCZs. As LCZ is a classification concept, we used the area proportion of each LCZ to quantify the LCZ. Finally, we used correlation and regression analyses to measure the effects of different LCZs on warming and cooling, thereby showing their combined effect. The ranking of the contribution of different LCZs to LST was compared by a standardized coefficient. This study aimed to solve two problems: (1) Creating a new method to measure the combined impact between LCZs, and (2) revealing the thermal characteristics of LCZ under combined effects. We used the area within the fourth ring road of Shenyang as a case study, but this method can be extended to other areas. Our results can explain the relationship between LST and LCZ, identify the combined influence of different types of LCZs on the urban thermal environment, and provide a reference for urban renewal, planning, and future construction.

## Data and methods

### Study area

**Figure 1:**
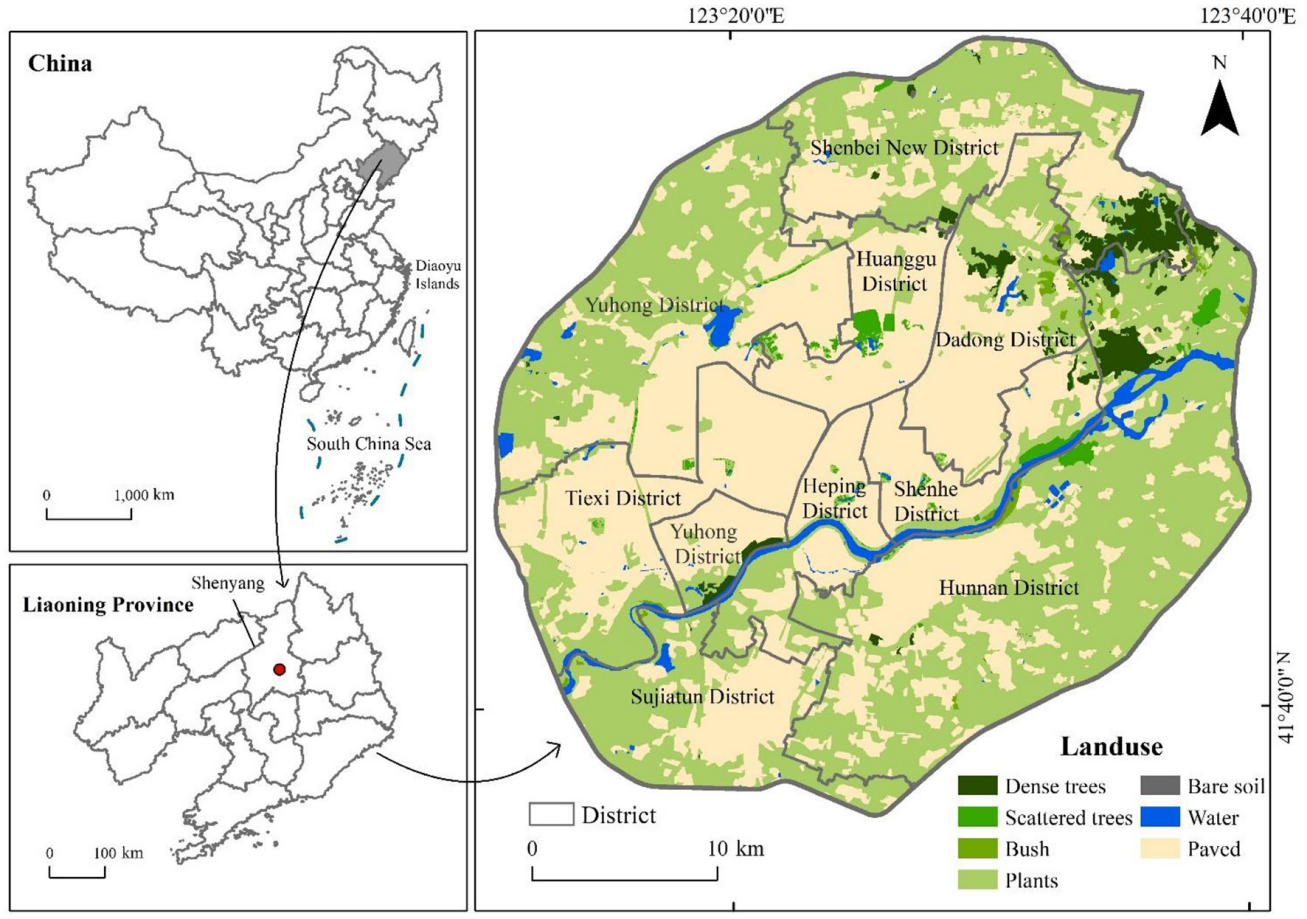
Location of the study area.

The study area is located in Shenyang, the capital of Liaoning Province, in northeastern China (122°25′-123°48′E, 41°12′-43°2′N). The terrain is relatively flat and mainly consists of plains. The eastern boundary extends from the hills of eastern Liaoning, and the western region comprises the alluvial plain formed by the Liao and Hun rivers. Shenyang has four distinct seasons and a temperate semi-humid continental climate with an annual average temperature of 6.2–9.7°C. Precipitation occurs mostly in summer, often in the form of torrential rains in July and August. The study area is within the Fourth Ring Road of Shenyang (Figure 1), which is the central urban area of socio-economic development in Shenyang. To balance the processes of urban development and the construction of human settlements, it is essential to study the thermal environment.

### Data source

The data includes land use, Landsat-8 images, MODIS images, building vectors, and other auxiliary data. The detailed attributes and sources are listed in Table 1. A land use and cover change data including secondary classification is used. The land use data in this study have been recorded every 5 years. We used one of its pictures in 2015. To ascertain the actual land use in the study area, the land use data of 2015 were partially updated using the multi-spectral image data of Landsat-8 in 2018. Using manual visual interpretation, the green space contour of some large parks in Shenyang was extracted. Finally, potential features classified by LCZ are reclassified into seven types in the figure. The updated land use data are shown in Figure 1.

**Table 1:** Research data and sources.

**Data**	**Description**	**Source**
Raster	Land use and cover change data, (30 m, 2015)	www.resdc.cn
Remote sensing data	MOD11A2 Ts production, 1,000 m, (28 July, 2018, 13 August, 2018)	USGS
	Landsat-8, (30 m, 12 August, 2018)	gscloud.cn
Building data	Building profile data, (2018)	map.baidu.com
Auxiliary data	Study area vector data	webmap.cn

### Method

**Figure 2:**
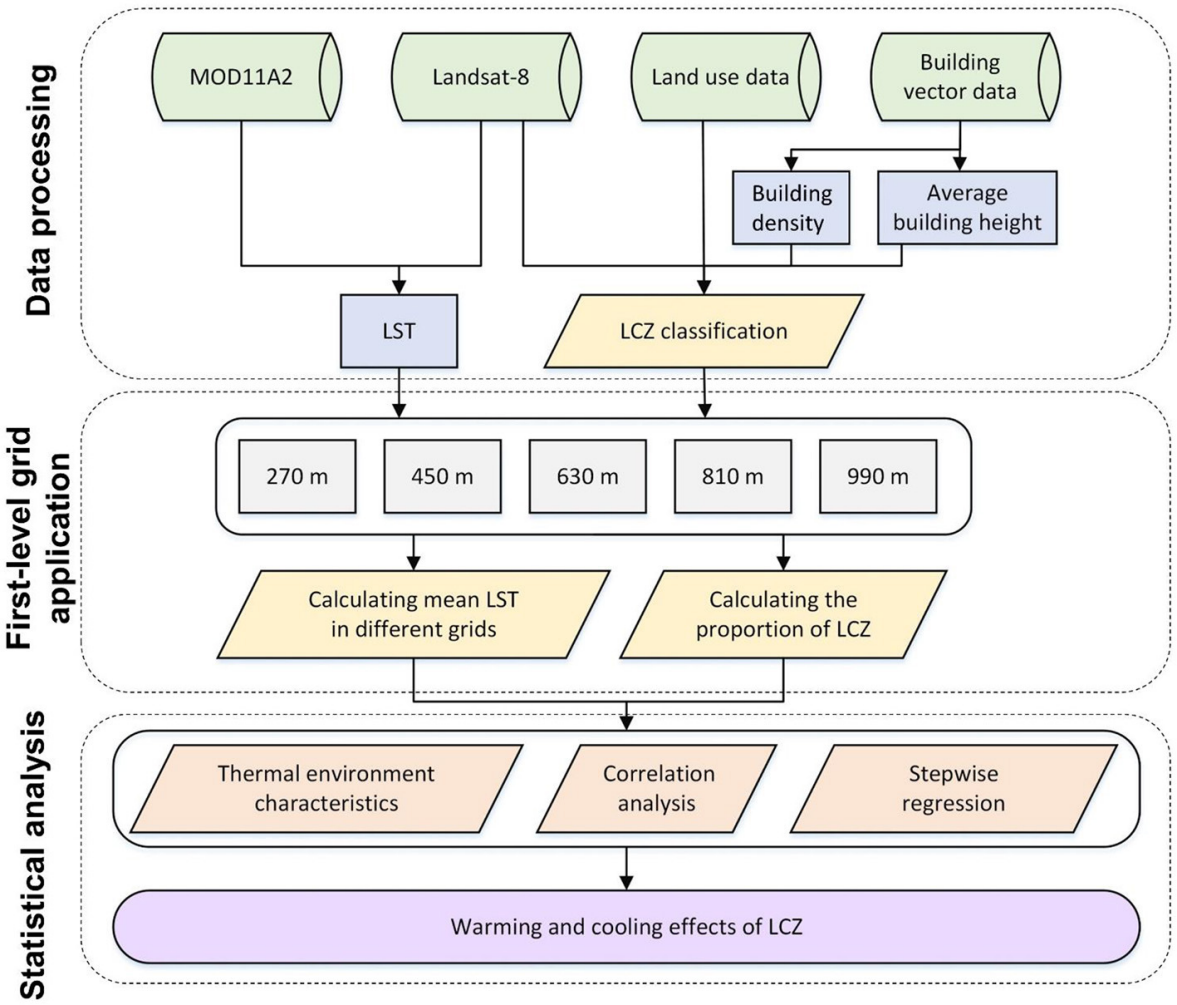
Research framework.

We used remote sensing data, land use data and building vector data to calculate LST and divide LCZs. The concept of first-level grid was introduced to calculate the area proportion of LCZ. The correlation was calculated for area proportion of LCZ and LST. The stepwise regression model was used to analyze the warming and cooling effects of LCZ under the combined influence. The frame figure is shown in Figure 2.

#### LCZ classification

We combined land use and building data to classify LCZs. A 30 m grid was established for the study area, and the BD and average BH of each grid were calculated. The classification criteria are shown in Table 2 (33, 35, 38, 52). The LCZ was classified into six building coverage types comprising LCZ1–LCZ6, and seven non-building coverage types (i.e., natural surface type) comprising LCZA–LCZG.

**Table 2:** Classification criteria of LCZs.

**LCZ**	**Description**	**LCZ**	**Description**
LCZ1	Compact high-rise	LCZA	Dense trees
LCZ2	Compact middle-rise	LCZB	Scattered trees
LCZ3	Compact low-rise	LCZC	Bush, scrub
LCZ4	Open high-rise	LCZD	Low plants
LCZ5	Open middle-rise	LCZE	Bare rock or paver
LCZ6	Open low-rise	LCZF	Bare soil or sand
		LCZG	Water

#### LST calculation

In this study, the LSTs were obtained from Landsat-8 and MODIS data. We used the single-window algorithm for retrieving LST from Landsat-8 TIRS10 images (53, 54). Using Planck's formula, the LST calculated using Landsat-8 T_S is as per formula (1):


(1) $$\begin{array}{l} T_{s}=\frac{K_{2}}{ln\left(1+\frac{K_{1}}{B\left(T_{S}\right)}\right)} \end{array}$$

*B*(*T*_*S*_) represents black body radiance, *K*_1_ = 774.89, and *K*_2_ = 1,321.08.

Further, to eliminate the influence of outliers that may exist in single-day data, the average was calculated using synthetic MOD11A2 (acquired on 28 July and 13 August, 2018). MOD11A2 is an 8-day composite LST product of MODIS, including daytime and nighttime data. We select daytime data whose collection time is consistent with Landsat-8. MOD11A2 was selected and all data were resampled to 30 m resolution. Finally, the average value of Landsat LST and MODIS LST was calculated as the ultimate LST of the study area in August, with a resolution of 30 m.

#### Thermal contribution calculation

**Figure 3:**
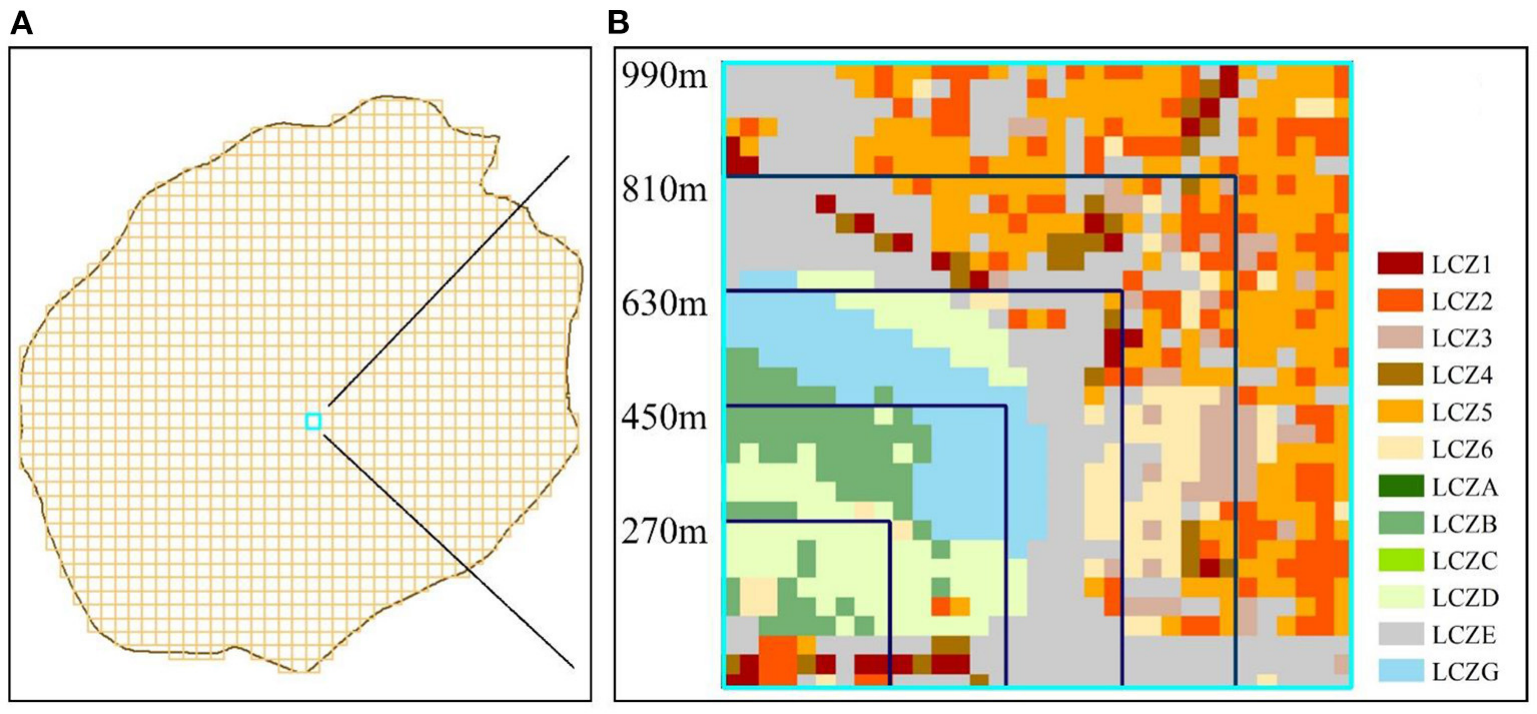
Division of the first-level grid; **(A)** 990 m grid covering the study area. **(B)** Number of LCZ covered by grids of different scales.

Based on the method of calculating the area proportion of land use types, this study established a numerical relationship between the area proportion of LCZ and LST (51). To quantitatively represent the LCZ and study the relationship between different LCZs, five levels of first-level grids were created as statistical units (50). The first-level grid refers to the basic unit used to calculate the area proportion of LCZ. Within the scope of each first-level grid, the total area proportion of various LCZs is 1, and can be regarded as the impact of the competition between LCZs on the LST of the first-level grid. The combined effect is shown as the warming and cooling effect of LCZ. Therefore, the concept of first-level grid can be used to assess the combined effect of LCZ on LST. It remains uncertain which scale the combined effect of LCZ is more significant, hence this study used a variety of scales of the first-level grid. The cell sizes of the grids were 270, 450, 630, 810, and 990 m. Figure 3 shows the case of a 990 m grid covering the study area. When a first-level grid was located covering the entire study area as shown in Figure 3A, the sample was adopted. The total qualified grids were: 16,624, 5,904, 2,977, 1,776, and 1,177. Figure 3B shows the LCZ range covered by the five first-level grids. We then calculated the proportions of various LCZs and average LSTs under the first-level grid cells using ArcGIS 10.8 tools, such as intersection and summarization.

Next, Pearson correlation analysis was conducted to investigate the presence of significant correlations between LCZ variables and LST. A stepwise regression model was established to analyze the combined impact of different LCZ and LST types and the contribution of each type of LCZ to LST. The stepwise regression model can select the most important variables and analyze the specific dependence between independent variables (the area proportion of LCZ) and dependent variables (LST). This stepwise regression tested the significance and contribution by introducing independent variables individually and removing independent variables that did not meet the standard. We used the *F*-value to express the conditions for entering the independent variable model. When *F* < 0.05, the independent variable can enter the model, while *F* > 0.1 indicates that the independent variable is removed. The stepwise regression model was constructed using IBM SPSS statistics 26. The process was repeated until no independent variables remained to enter or be removed from the model (55). The best linear regression model was established by considering LST as the dependent variable and LCZ area proportion as the independent variable. In this case, all variables in the regression model were significant. The final regression model was as follows:


(2) $$\begin{array}{l} LST=\beta_{1}\times LCZX_{1}+\beta_{2}\times LCZX_{2}+\;+\beta_{n}\times LCZX_{n}+b\;\cdot \end{array}$$

where LCZ*X*_*n*_ represents the proportion of LCZ1–G; β_*n*_ is the regression coefficient of LCZ*X*_*n*_; *b* is a constant term.

We normalized β_1_− β_*n*_ to compare the contribution of different types of LCZ to the LST. The standardized coefficients were used to measure the direction and relative size of the contribution of different LCZs to the LST at different scales. A positive standardized coefficient indicates that the LCZ type has a warming effect on the LST, and a negative standardized coefficient indicates a cooling effect. The higher the value, the higher the actual contribution rate of the LCZ type to the LST under the same area proportion.

## Results

### Thermal environment characteristics from the LCZ perspective

**Figure 4:**
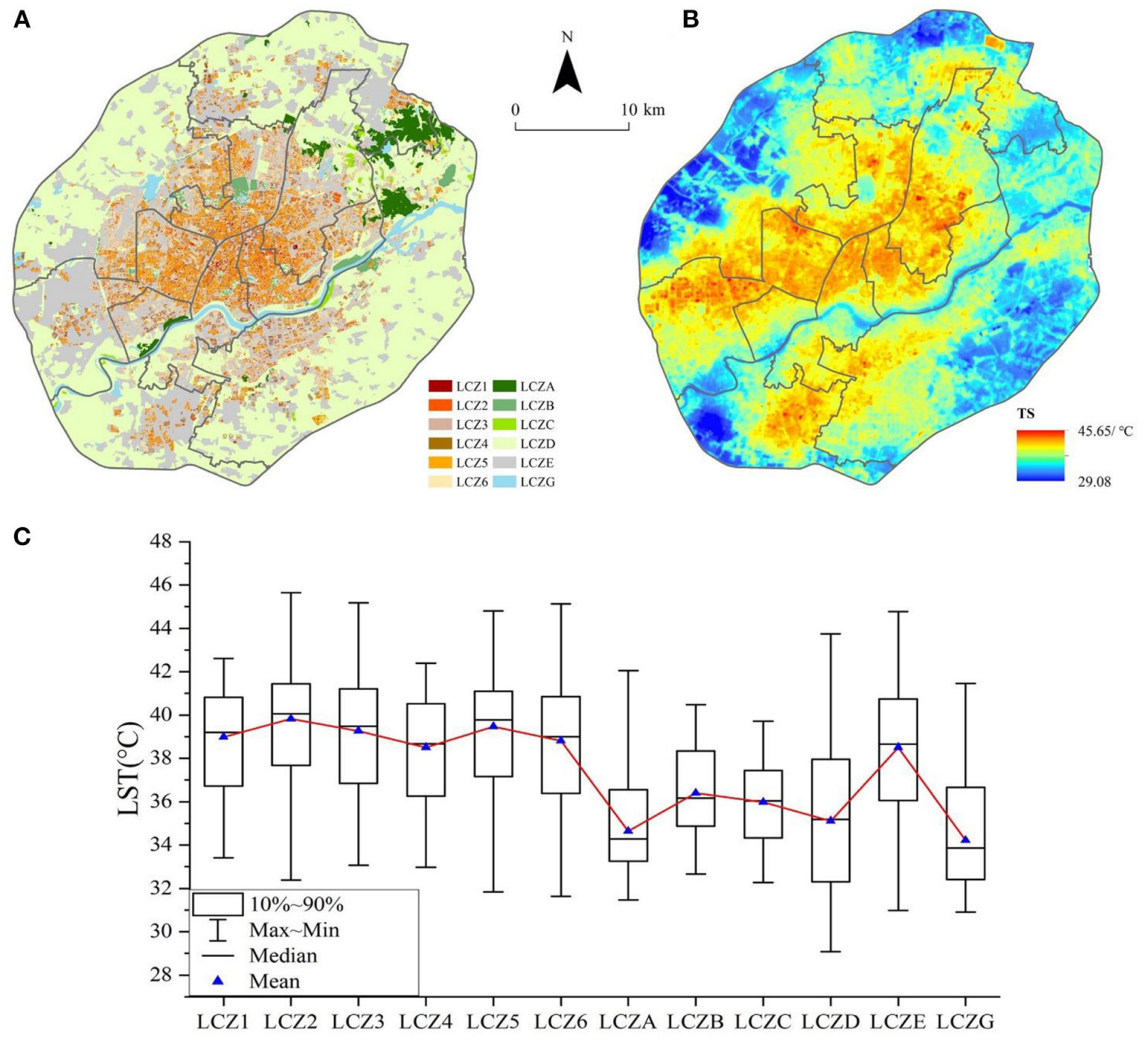
Thermal characteristics of the LCZ; **(A)** LCZ classification results; **(B)** Inversion results of land surface temperature in the study area; **(C)** Boxplot of LST distribution under LCZ.

LCZ classification results of the study area are shown in Figure 4A. The study area contained 12 of the total 13 LCZ types, excluding the category of LCZF (bare soil or sand). Overall, for the construction area: the overall proportion of LCZ1–LCZ6 was 19.3%, and the overall proportion of LCZA–LCZG was 80.7%. The proportion of all LCZs, from high to low, was as follows: low plants LCZD (44.4%) > bare rock or paver LCZE (30.0%) > open middle-rise building LCZ5 (5.8%) > open low-rise building LCZ6 (5.0%) > compact low-rise building LCZ3 (3.5%) > compact middle-rise building LCZ2 (3.2%) > water LCZG (2.5%) > dense trees LCZA (2.4%) > open high-rise building LCZ4 (1.2%) > scattered trees LCZB (1.0%) > compact high-rise building LCZ1 (0.6%) > bush and scrub LCZC (0.4%).

The calculated LSTs of the study area are shown in Figure 4B. In the summer of 2018, the LST in the urban area within the Fourth Ring Road of Shenyang was between 29.08 and 45.65°C, and the average LST was 36.88°C. Approximately 95% of the pixels were at temperatures within the range of 31.54–41.2°C, with few extreme temperature pixels.

Figure 4C shows the temperature characteristics of various LCZs. In the LCZ category of the built-up area, the average surface temperature (TsMean) was higher than 38°C. The LCZ type with the highest TsMean was LCZ2 compact middle-rise building (39.82°C) and that with the lowest TsMean was LCZ4 open high-rise building (38.51°C). The highest statistical range of the inter-class mean surface temperature in the LCZ of the built-up zone was 13.5°C, corresponding to LCZ6. In the natural area LCZs, the highest TsMean type was LCZE (38.51°C) and the lowest was LCZG water body (34.21°C), which also had the lowest average surface temperature among all LCZ types.

In the case of building class LCZ, the low- and middle-rise building classes (LCZ2, LCZ3, LCZ5, and LCZ6) had maximum and average temperatures higher than that of the high-rise buildings (LCZ1 and LCZ4) because tall buildings tend to cast shadows, which reduces the surface temperature; this is consistent with the findings of other studies (56). However, the minimum temperature of the high-rise buildings was higher than that of the middle- and low-rise buildings, under the corresponding BD, which may be due to the relatively high warming effect of LCZ types with high average BHs. The LCZ type with a high BD contributes more to the LST, and the warming effect is more pronounced.

### Correlation between LCZ proportion and LST

#### Calculation of LCZ proportion at different grid scales

We calculated the proportion of different LCZ types in each grid. Table 3 lists the quantitative characteristics of the LCZ area proportion calculated under the first-level grids of different scales. As the area and spatial distribution patterns of different LCZ types were different, the area proportion calculated for some first-level grids might be zero. In Table 3, the first line represents the actual number of grids where the calculated area proportion was greater than zero. The second line represents the average area proportion after removing the zero terms.

**Table 3:** The quantitative characteristics of the LCZ area proportion.

**Type**	**270 m**	**450 m**	**630 m**	**810 m**	**990 m**	**Implication**
LCZ1	2,086	1,234	824	595	454	Valid item count
	0.050	0.030	0.023	0.019	0.017	Average area proportion
LCZ2	4,803	2,270	1,372	938	694	
	0.113	0.086	0.072	0.064	0.058	
LCZ3	5,635	2,693	1,550	1,023	735	
	0.105	0.079	0.070	0.064	0.060	
LCZ4	2,462	1,375	905	633	483	
	0.079	0.051	0.039	0.034	0.029	
LCZ5	5,296	2,459	1,452	993	722	
	0.185	0.143	0.123	0.109	0.100	
LCZ6	6,656	2,958	1,679	1,086	771	
	0.128	0.104	0.093	0.087	0.082	
LCZA	833	411	257	198	142	
	0.482	0.349	0.279	0.219	0.203	
LCZB	623	352	254	187	157	
	0.279	0.177	0.126	0.102	0.082	
LCZC	204	121	79	56	46	
	0.356	0.216	0.164	0.141	0.111	
LCZD	10,368	4,127	2,225	1,381	957	
	0.701	0.619	0.574	0.546	0.521	
LCZE	10,499	4,283	2,357	1,539	1,061	
	0.479	0.419	0.386	0.354	0.341	
LCZG	982	521	357	264	213	
	0.424	0.284	0.210	0.169	0.139	

**Figure 5:**
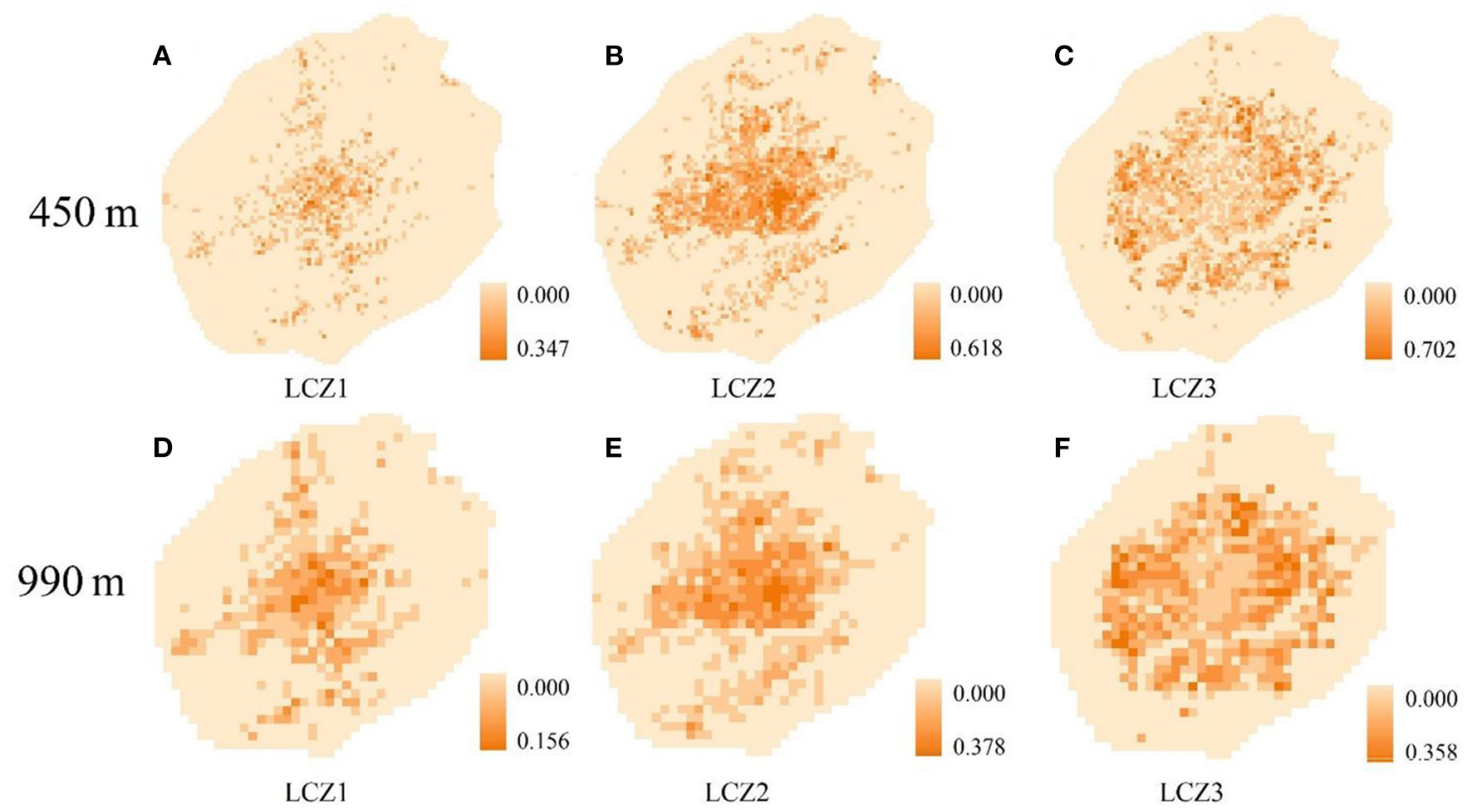
Proportion of LCZs in different scales; **(A–C)** distribution of LCZ1–3 at a scale of 450 m; **(D–F)** distribution of LCZ1–3 at a scale of 990 m.

For example, the area proportion of LCZ1–3 at scales of 450 and 990 m are shown in Figure 5. With the increase in grid scale, the maximum ratio of LCZ1–3 gradually decreased. At the 450 m scale, the maximum ratio of LCZ1 was 0.347; at the 990 m scale, it decreased to 0.156.

#### Correlation analysis

**Figure 6:**
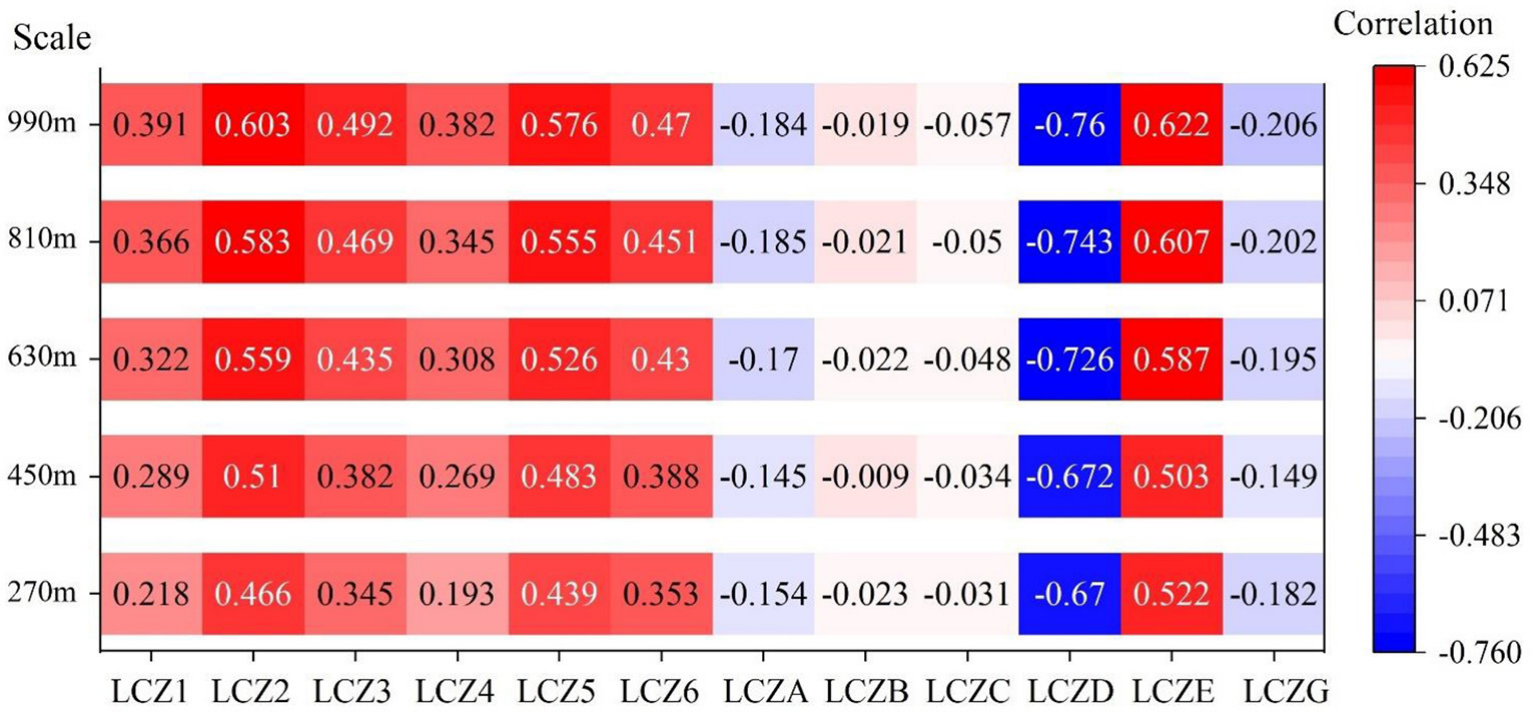
Correlation coefficients between LCZ and LST at different first-level grid scales.

The correlation between the LCZ area proportion and the corresponding mean LST at each scale is shown in Figure 6. For built-up zones, the area proportions of LCZ1–6 showed a significant positive relationship with LST at all scales. Among them, LCZ2 showed the highest correlation, followed by LCZ5; LCZ4 showed the lowest correlation. This is consistent with the average temperature characteristics described in section 3.1. For natural areas, LCZA, LCZD, and LCZG showed significant negative correlations with LST, with LCZD having the strongest negative correlation. Among the natural area LCZs, LCZE was the only variable that showed a significant positive correlation with LST. The correlations of LCZB and LCZC with LST were weak with low significance. Therefore, in further discussions, the contributions of LCZB and LCZC to the LST and their combined effect will not be considered. The correlations of other variables were significant at 0.01 level.

With the increase in first-level grid scales, although the maximum proportion of built-up area gradually decreased, the correlation between the maximum area proportions of LCZ1–6, and LST showed an increasing trend. For natural area LCZA–G, the correlation increased with increasing grid scale. However, at 450 m, the correlation of LCZA, LCZE, and LCZG showed a decreasing trend. In built-up area LCZs, the correlation between LCZ and LST was more affected by grid scale.

### LCZ thermal contribution ranking analysis

#### Parameters and adjusted *R^2^* values of optimal stepwise regression model

According to the results of the correlation analysis, 10 variables were selected for a stepwise regression analysis, which included LCZ1, LCZ2, LCZ3, LCZ4, LCZ5, LCZ6, LCZA, LCZD, LCZE, and LCZG. At each scale, the final independent variables and their adjusted *R*^2^ values used in the model are shown in Table 4.

**Table 4:** Adjusted *R*^2^ of stepwise regression model.

**Scales**	**Input variables (listed in order of entry)**	**Adjusted *R*^2^**
270 m	LCZD, LCZG, LCZA, LCZ2, LCZ3, LCZE, LCZ5, LCZ6, and LCZ1	0.608
450 m	LCZD, LCZA, LCZG, LCZ2, LCZ3, LCZE, LCZ5, LCZ6, and LCZ1	0.576
630 m	LCZD, LCZA, LCZG, LCZ2, LCZ3, LCZE, LCZ5, and LCZ6	0.680
810 m	LCZD, LCZA, LCZG, LCZ2, LCZ3, LCZE, LCZ5, and LCZ6	0.704
990 m	LCZD, LCZA, LCZG, LCZ2, LCZ3, and LCZE	0.724

The first-level grids of different scales are used to measure the sensitivity of the combined effects of different types of LCZs to changes in distance. This limits the influence range of each LCZ grid from the geographical distance. When the primary grid scale is small, most LCZs can significantly affect the LST, which shows that there are more variables entering the model. With the increased first-level grid scale, the influence scope of some LCZs becomes smaller, which means that they cannot enter the stepwise regression model. As the grid scale increases, the number of independent variables entering the model decreases. At 270 and 450 m scales, 9 LCZs were included in the optimal model. At 630 and 810 m scales, there were 8 LCZ types included in the model. However, there were only 6 types at 990 m. Although LCZ4 showed a significant positive correlation with LST, it failed to enter the model at all scales, indicating that the current LCZ4 distribution in Shenyang cannot significantly increase or decrease the LST. Similarly, with the increase in grid scale, the number of LCZ types that can affect the LST in Shenyang gradually decreases. The adjusted *R*^2^ value of the regression model was >0.5 at all scales after considering the combined effects of the LCZs, and the interaction between them. Using the proportion of LCZ within a fixed range as an independent variable can explain more than 50% of the variation in LST of the dependent variable. The adjusted *R*^2^ of the regression models at different scales listed in Table 4 shows that overall, the larger the first-level grid, the greater the model significance, and the higher the explanatory power of the LCZ for surface temperature changes. At the 270 m scale, the adjusted *R*^2^ of the model was 0.608; it increased to 0.724 at the 990 m scale, increasing the explanatory power by 19.1%. At the 450 m scale, the adjusted *R*^2^ was less than all other scales.

#### Effect of LCZ warming and cooling

**Figure 7:**
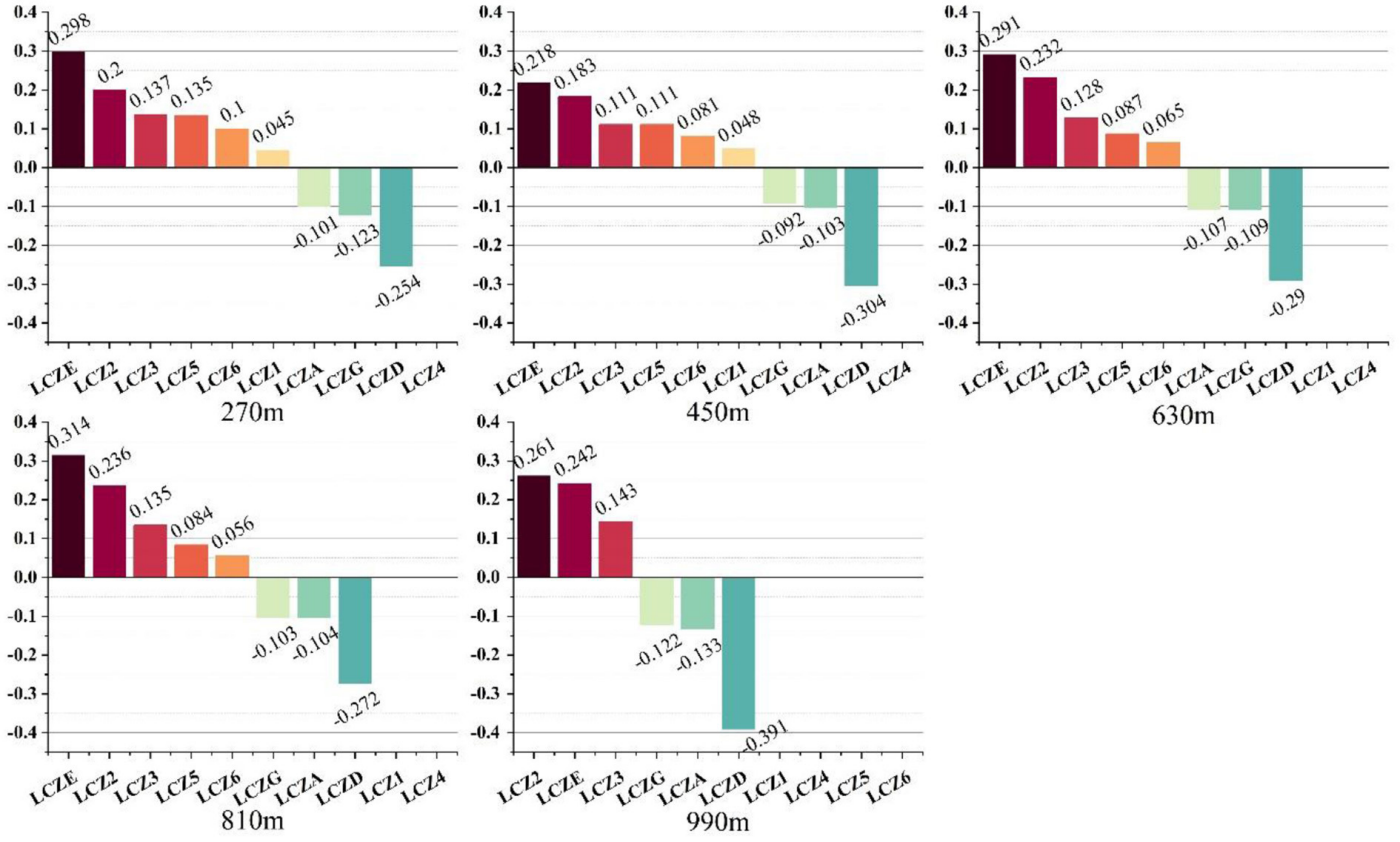
Normalized coefficients of various LCZ and LST regression models under the combined influence.

The contribution of various LCZs to LST can be quantitatively compared by comparing the magnitude of the standardized coefficients. Thus, we can easily analyse which LCZs have significant impacts on the LST changes, and the direction of their impact at different grid scales, as shown in Figure 7. Overall, the LCZ types that played a positive role in warming were LCZE, LCZ2, and LCZ3. The LCZs that played a negative cooling role were LCZD, LCZA, and LCZG. LCZ1, LCZ5, and LCZ6 increased the temperature by small scales, such as 270 m. However, with the increase in grid scale, their warming effect was not significant in the model, therefore they were excluded.

At different grid sizes, the heating and cooling effects of LCZ were different. For example, at the 270 m scale, the warming effect from high to low was LCZE (0.298) > LCZ2 (0.200) > LCZ3 (0.137) > LCZ5 (0.135) > LCZ6 (0.100) > LCZ1 (0.015). At the 990 m scale, the warming effect of LCZ2 exceeded that of LCZE. Only three LCZs significantly affected the increase of LST: LCZ2 (0.261) > LCZE (0.242) > LCZ3 (0.143). At the 270 m scale, the cooling effect was in the order of: LCZD (−0.254) > LCZG (−0.123) > LCZA (−0.101). However, at other scales, the order of cooling effect was LCZD > LCZA > LCZG.

In our study, 810 m was the most suitable scale for the calculation of LCZ contribution in Shenyang. When *R*^2^ was >0.7, we considered that the model was reliable. The calculation of contribution requires that the simultaneous action of as many LCZ types as possible is considered, as the number of effective independent variables entering the model is important. An increase in scale leads to a decrease in the number of independent variables entering the model. When the model scale increased from 810 to 990 m, only 6 LCZ types could be considered. This means that 10.8% of the LCZ area was ignored but *R*^2^ was only improved by 3.5%. Therefore, the 810 m scale, at which the model showed a high adjusted *R*^2^ value with more independent variables entering the model, was considered the best scale. At 810 m, the warming effect was in the order of: LCZE (0.314) > LCZ2 (0.236) > LCZ3 (0.135) > LCZ5 (0.084) > LCZ6 (0.056). The cooling effect was in the order of: LCZD (−0.272) > LCZA (−0.104) > LCZG (−0.103). The results show that the method of calculating the area ratio of LCZ and incorporating it into the stepwise regression equation can measure the combined influence of LCZ and explain about 70% of LST changes. At the same time, compared with the traditional perspective, the contribution of LCZ considering the combined impact to the surface temperature is different. This shows that it is necessary to consider the actual contribution of the LCZ under the combined influence.

## Discussion

### Interaction between LCZs

To establish the relationship between LCZ and the thermal environment, the average temperatures within LCZs are usually used to estimate the thermal characteristics (32). We also applied this method to analyze the thermal environment characteristics of Shenyang in summer, as described in section 3.1. Previous studies show that many factors within the LCZ can be quantified, such as NDVI, BH, the proportion of impervious water surface, and the average tree height using this method (26, 41). These studies elucidated the influence of internal characteristics of LCZ on LST. However, they estimated the impact of each type of LCZ on LST in an independent state. Therefore, when the correlation between LCZ1 and LST is analyzed, the area and location of other LCZs, such as LCZ2 and LCZB, do not have any impact on the correlation results. But in fact, LCZs do not exist independently in the city; they are adjacent to, or intersect with, other LCZs. Therefore, an important but unanswered question was whether LCZs had combined or competitive influence on LST. To answer this question, we proposed to use the area proportion to describe LCZs. In this method, we demarcated a series of first-level grids and calculated the area proportion of LCZs.

As the area proportion is competitive, all LCZs are no longer independent LST-related variables, but their changes are closely related to other LCZs. They are placed in an environment with a total of “1,” competing for influence on the LST. The results of the calculated area proportion are described in section 3.2. However, the relevant analysis method is independent and does not reflect the competition and combined influence of LCZs. Therefore, the ranking of thermal characteristics measured by average temperature is consistent with the correlation. As all LCZs were included in the regression model, they required a simultaneous impact on LST, and the results changed. We found that the warming effect of LCZE and the cooling effect of LCZG might have been underestimated due to the neglect of the combined and competitive effects of LCZs in the past. It should be noted that these results apply only to the current LCZ configuration in Shenyang. However, the method of using area proportions to analyze the combined effect of LCZs on LST can be applied to other regions to explore the factors that have been neglected in the independent analysis.

### A new perspective on understanding urban thermal environment

We can find that the influence of different LCZ categories on the LST, calculated by a stepwise regression (section 3.3), is not completely consistent with their thermal characteristics (i.e., the corresponding average LST of each LCZ category; section 3.1), or the degree of correlation (section 3.2). The average temperature ranking of LCZ was a description from a regional perspective not involving the interaction between LCZ categories. The combined effect emphasized that LCZ impacts LST at the same time, considering the interaction between different categories. The differences reflected from the two perspectives suggest the presence of combined effect. This may be associated with the change in heat storage capacity caused by the arrangement of LCZ. Although LCZ2 has the highest mean surface temperature, the regression results show that LCZE has a higher warming effect on LST than LCZ2 across the four scales from 270 to 810 m. Previous studies focused on the heat generated by building coverage LCZs to the urban thermal environment. The strong warming effect produced by LCZE has not been discussed. Meanwhile, despite the low surface temperature of LCZG, it failed to produce a sufficient cooling effect in the stepwise regression model. A different perspective on the LST contribution can explain this contradiction. The average temperature of an LCZ shows its thermal state, which cannot be used to estimate the actual contribution to urban surface temperature because its area and distribution are not considered. The standardized coefficient obtained by stepwise regression modeling represents a relative state and is used to calculate the degree of LST change caused by LCZ. Combining the two factors can better analyze the urban thermal environment. The cooling of the city alone cannot explain the local minimization; instead, it is essential to consider the combined influence of various factors to achieve the optimal effect.

### Strategies for cooling cities

To achieve the goal of urban cooling, many studies have been conducted on land use adjustment, planning, and construction (15, 57). For example, in China's Beijing-Tianjin-Hebei, Pearl River Delta, and Yangtze River Delta urban agglomerations, the LST of the compact medium- and high-rise buildings is high, which is not conducive to urban cooling (40). However, green spaces and water bodies can be added to compact high-rise buildings to meet the cooling requirements (58). Optimizing ventilation corridors and increasing the area of green space and water bodies can cool the city (59–61).

This study provides some new cooling ideas based on the contribution of LCZ to LST. The thermal characteristics (i.e., average LST) and their ranking based on temperature-increasing ability (i.e., standardized coefficient) of different LCZs were determined. For LCZ types with high average temperatures, such as building-type LCZs and LCZE, measures should be taken to reduce their LST. Increasing the greening between buildings and optimizing roof materials or ground paving materials may play a cooling role. To alleviate the influence of high-temperature, reduce the LST, and achieve the purpose of cooling the city, we should explore the cooling potential of trees in the city (62, 63). For LCZs with a strong warming effect, such as LCZ2, LCZ3, and LCZE, the excessive increase should be avoided in the process of urban planning. Simultaneously, a database on the variation of the warming effect of different LCZs in cities should be established. The thermal characteristics and warming effect of LCZs should be recorded and analyzed regularly to provide a reference for the thermal environment background of future urban construction.

For the study area, the main central high temperature area is continuous. This is similar to the distribution trend of LCZE. Therefore, in the future urban planning of Shenyang, we proposed building greenways, increasing LCZD and reducing the combined warming effect caused by impervious surface. On a smaller scale, especially in the built-up areas of the city center, including Shenhe, Heping, and Huanggu Districts, we propose the construction of pocket parks to improve the cooling capacity.

### Limiting factors

To facilitate the calculation of LCZ area proportion, we preferentially choose the 30 m LCZ division, which may cause the spatial discontinuity of LCZs. With respect to the classification of LCZs in this study, there is still room for the improvement in classification accuracy owing to the timeliness and availability of data (64). Results can be improved by optimizing the LCZ classification and area proportion calculation methods.

Using only the area proportion as a measure of LCZ cannot reflect the spatial location attributes of LCZ and the intra-type differences of LCZ. Our study could not reveal whether the spatial distribution of LCZs affects LST, which requires some new models or perspectives to be considered, such as the geographic weighted regression model and landscape pattern index (65, 66).

Finally, although we could quantify the relative contribution, the causes have not been identified. It may still be necessary to consider the mutual relationship and competitive impact between LCZs, which will be addressed in future research.

## Conclusion

This study analyzed the spatial distribution characteristics of LST based on different types of LCZs in Shenyang City. The proposed method used stepwise regression analysis to quantify the contribution of LCZ area proportion to LST. The main conclusions are as follows:

The study area was classified into 30 m LCZs. The LCZs of the building and natural areas accounted for 19.33 and 80.67% of the units, respectively. The proportion of natural species was the highest in LCZD (44.34%) and lowest in LCZC (0.4%). Construction accounted for the largest proportion of units in LCZ5 (5.8%) and the least in LCZ6 (0.6%). In terms of spatial distribution, construction LCZ presented a spatial pattern of staggered intermixed distribution, whereas natural LCZ was more concentrated and independent. There was a significant heat island effect in the study area. The high-temperature area was concentrated in the urban built-up area, and the average LST was 36.88°C. The highest average LST of 39.82°C was observed in a LCZ2 middle-level compact building and the lowest average LST of 34.24°C was observed in the LCZG water body.

Five first-level grids with different scales were established to calculate the area proportion of LCZ. The LCZ1–6 and LCZE showed a significant positive correlation with LST, while LCZA, LCZD and LCZG showed significant negative correlations with LST. LCZB and LCZC showed weak correlations with LST. With the increase in the first-level grid scale, the correlation between LCZ and LST generally exhibited an upward trend. In the case of building areas, the correlation between LCZ and LST was more affected by grid scale.

From 270 to 990 m, the LCZs entering the stepwise regression model were different. As the grid scale increased, the independent variables entering the model decreased. The adjusted *R*^2^ values of stepwise regression models at different grid scales were 0.608 (270 m), 0.576 (450 m), 0.680 (630 m), 0.704 (810 m), and 0.724 (990 m). The influence of LCZ on LST was determined by calculating the standardized coefficients. The most suitable scale for the calculation of LCZ contribution in Shenyang was 810 m. The warming effect was in the following order: LCZE (0.314) > LCZ2 (0.236) > LCZ3 (0.135) > LCZ5 (0.084) > LCZ6 (0.056); the cooling effect showed the following order: LCZD (−0.272) > LCZA (−0.104) > LCZG (−0.103). Unlike the average LST, this method determined the effect on the LST change considering the interaction between LCZs.

## Data availability statement

The original contributions presented in the study are included in the article/supplementary material, further inquiries can be directed to the corresponding authors.

## Author contributions

RZ: data curation, software, and writing—reviewing and editing. JY and DS: conceptualization, methodology, and reviewing and editing. XM, XX, and JX: writing—reviewing and editing. WY: data curation and reviewing and editing. All authors contributed to the article and approved the submitted version.

## Funding

This research study was supported by the National Natural Science Foundation of China (Grant no's 41771178, 42030409), The Fundamental Research Funds for the Central Universities (Grant no. N2111003), and Basic Scientific Research Project (Key Project) of the Education Department of Liaoning Province (Grant no. LJKZ0964).

## Conflict of interest

The authors declare that the research was conducted in the absence of any commercial or financial relationships that could be construed as a potential conflict of interest.

## Publisher's note

All claims expressed in this article are solely those of the authors and do not necessarily represent those of their affiliated organizations, or those of the publisher, the editors and the reviewers. Any product that may be evaluated in this article, or claim that may be made by its manufacturer, is not guaranteed or endorsed by the publisher.
